# Upregulation of tRNA-Ser-AGA-2-1 Promotes Malignant Behavior in Normal Bronchial Cells

**DOI:** 10.3389/fmolb.2022.809985

**Published:** 2022-05-02

**Authors:** Mafalda Santos, Ana Fidalgo, Ana Sofia Varanda, Ana Raquel Soares, Gabriela M. Almeida, Diana Martins, Nuno Mendes, Carla Oliveira, Manuel A. S. Santos

**Affiliations:** ^1^ Expression Regulation in Cancer, Instituto de Investigação e Inovação em Saúde, University of Porto, Porto, Portugal; ^2^ Institute of Molecular Pathology and Immunology University of Porto (IPATIMUP), Porto, Portugal; ^3^ Department of Medical Sciences, Institute of Biomedicine – iBiMED, University of Aveiro, Aveiro, Portugal; ^4^ Department Pathology, Medical Faculty of Porto, Porto, Portugal

**Keywords:** tRNA deregulation, cancer, tumor establishment, UPR, lung cancer

## Abstract

Serine tRNAs (tRNA^Ser^) are frequently overexpressed in tumors and associated with poor prognosis and increased risk of recurrence in breast cancer. Impairment of tRNA biogenesis and abundance also impacts proteome homeostasis, and activates protein quality control systems. Herein, we aimed at testing whether increasing tRNA^Ser^ abundance could foster tumor establishment through activation of the UPR. In order to do so, firstly we confirmed that the expression of tRNA-Ser-AGA-2-1 [hereafter tRNA^Ser^(AGA)] was upregulated by 1.79-fold in Stage I NSCLC tumors when compared to normal adjacent tissue. To study the impact of tRNA^Ser^(AGA) in early stage tumorigenesis, we induced its upregulation in a non-tumoral bronchial cell line, BEAS-2B. Upregulation of this tRNA increased cellular proliferation and protein synthesis rate, driven by eIF2*α* dephosphorylation and ATF4 activation downstream of PERK signaling. Futhermore, tRNA^Ser^(AGA) enhanced transformation potential *in vitro*, and promoted the establishment of slow growing tumors with aggressive features in nude mice. Our work highlights the importance of studying tRNA deregulation on early stage tumorigenesis, as they may be potential malignancy and aggressiveness biomarkers.

## Introduction

The expression levels of tRNAs that decode codons belonging to families of phosphorylation targeted amino acids, i.e., serine and threonine, are often found increased in tumors (Pavon-Eternod et al., 2009; Gingold et al., 2014), suggesting that tumors may require higher levels of certain tRNAs to sustain their proliferation. This hypothesis is corroborated by the finding that global tRNA profiles change significantly in tumors, and that such altered profiles alone are sufficient to discriminate tumor samples from normal tissue, as it is the case for breast cancer (Krishnan et al., 2016). Moreover, the upregulation of tRNA^Glu^ (UUC) and tRNA^Arg^ (CCG) in breast cancer has been associated with increased translation of transcripts that are enriched in their cognate codons, i.e., Glu-GAA and Arg-CGG codons (Goodarzi et al., 2016). Overexpression of the methionine initiator tRNA (tRNA_i_Met) is associated with increased metabolic activity and proliferation of normal breast cells (Pavon-Eternod et al., 2013). This tRNA is able to rescue tumorigenic ability of breast cancer cell lines, in the presence of high levels of the tumor suppressor miR-34a (Wang et al., 2018), suggesting a role in breast tumor initiation. The overexpression of serine tRNAs (tRNA^Ser^) has been associated with poor prognosis of several types of cancer and increased risk of recurrence in breast cancer (Krishnan et al., 2016).

Our previous works uncovered a link between tRNA deregulation and the activation of the Unfolded Protein Response (UPR) and Ubiquitin-Proteasome system (UPS) both in mammalian cell lines and in tumors (Santos et al., 2018; Varanda et al., 2020), which may be explained by the possible increase in misfolded proteins (Yang et al., 2014). The PERK, ATF6, and IRE1α UPR branches normally activate a pro-survival cellular response, but stress persistence leads them to switch into a pro-apoptotic signaling mode (Lu et al., 2014). Interestingly, cancer cells can hijack the UPR to increase tumor aggressiveness, tumor-promoting inflammation, survival during hypoxia and tumor resistance to treatment (Avril et al., 2017; Tam et al., 2012; Mujcic et al., 2013; Dejeans et al., 2015). This is particularly evident for the PERK branch of the UPR which controls protein synthesis rate, and prevents ER overloading with misfolded proteins (Harding et al., 2000a; Scheuner et al., 2001). Indeed, PERK phosphorylation of the initiation factor eIF2*α* inhibits the assembly of the translation initiation complex, leading to selective translation of stress response transcripts. This is the case of Transcription factor ATF4, CHOP and BiP, which counteract stress associated to tumor physiology (Harding et al., 2000b; Novoa et al., 2001). Decreased levels of eIF2*α* phosphorylation are sufficient to trigger transformation on a near-normal *Mus musculus* cell line (Perkins and Barber, 2004), but PERK can also activate known oncogenes such as Akt, mTOR and MAPK (Bobrovnikova-Marjon et al., 2008; Bobrovnikova-Marjon et al., 2010; Bu and Diehl, 2016). These data are supported by UPR activation in pre-malignant lesions of hepatocellular carcinoma (HCC), which, when combined with hypernutrition, lead to HCC development (Nakagawa et al., 2014).

In this work, we have extended previous studies on the role of tRNA expression deregulation in cancer by artificially increasing the expression of the human tRNA-Ser-AGA-2-1 in near-normal bronchial epithelium cells (BEAS-2B). Our data show that even a modest increase in tRNA^Ser^(AGA) abundance upregulates BiP and increases dephosphorylation of eIf2*α*, protein synthesis rate and cell proliferation. Overall, this work suggests that tRNA^Ser^(AGA) overexpression alone is sufficient to speed up the formation of slow growing tumors with increasing aggressiveness features.

## Materials and Methods

### tRNA Expression in Tumoral and Normal Lung Tissues

We assessed the tRNA-Ser-AGA-2-1 expression levels in both tumors and normal tissues using the table “Whole Expression at tRNA level (TMM)” available at the tRiC database (https://hanlab.uth.edu/tRic/download/). Next, we selected the Stage I and normal tissues based on the clinical data available at the GDC data portal (https://portal.gdc.cancer.gov/). A Student’s t-test was applied to infer statistical significance.

### Cell Culture

BEAS-2B cell line was kindly provided by Professor Maria Carmen Alpoim, from IBILI, University of Coimbra. BEAS-2B cells were cultured in LHC-9 medium (Gibco, Life Technologies) supplemented with 1% of Penicillin-Streptomycin (Pen/Strep) (Gibco, Life Technologies). NCI-H460 were obtained from the American Type Culture Collection (ATCC) and were cultured in RPMI 1640 medium (Gibco, Life Technologies), supplemented with 10% Fetal Bovine Serum (FBS) (Sigma) and 1% Pen/Strep. Cells were maintained in an incubator at 37°C with 5% CO_2_ and 95% relative humidity. To execute the following procedures, cells were detached using Tryple Xpress (Gibco, Life Technologies). Cells were regularly tested for *mycoplasma* contamination.

### Generation of Stable Cell Lines

BEAS-2B stable cell lines were generated through electroporation (three independent times) with the Mock and tRNA^Ser^(AGA) plasmids used in (Varanda et al., 2020). BEAS-2B cells were seeded in 100 mm dishes and cultured until 70–90% of confluence was reached. Cells were detached and the pellet was re-suspended in Hepes Buffered Saline (HBS) solution to improve the transfection efficiency. Then, 4 mm electroporation cuvettes were prepared with 10 µg of plasmid and 0.5 ml of cell suspension was added, mixing carefully. For each sample, the voltage applied was 230V, with capacitance of 1500 µF and resistance of 125*Ω*. This step was performed using ECM Electro Cell Manipulator (BTX, Harvard Apparatus). Immediately after the electroporation, 1 ml of LHC-9 culture medium (Gibco, Life Technologies) was added to the cuvette, cells were then carefully homogenized and transferred to 60 mm dishes. 5.0 × 10^4^ NCI-H460 cells were plated in MW24 plates, incubated for 48 h and then transfected using 1.5 μg of plasmid DNA and 0.75 μl of Lipofectamine^®^ 3,000 (Invitrogen). Three independent transfections were carried out for the empty vector (Mock). Stable cell lines were obtained by selection with either 200 μg/ml (BEAS-2B) or 800 μg/ml (NCI-H460) of G418 (Formedium) for 3 weeks.

### Extraction and Quantification of gDNA

To ensure that the recombinant plasmid did not acquire mutations upon integration in the genome, gDNA was extracted for Sanger sequencing. The NZY Tissue gDNA Isolation Kit was used following instructions recommended by the manufacturer. gDNA concentration was determined using a NanoDrop.

### RNA Extraction, cDNA Synthesis and tRNA Quantification

Total RNA was extracted using the NZY Total RNA Isolation kit (Nzytech, Cat. MB13402) according to the manufacturer’s instructions. The integrity and quantity of each RNA sample were assessed using a Denovix spectrophotometer (Denovix, Wilmington, DE, United States). 200 ng of total RNA were used for cDNA synthesis using NCode™ VILO™ miRNA cDNA Synthesis Kit (Invitrogen, Cat. A11193050), following the manufacturer’s instructions. PCR amplification of tRNA^Ser^ was performed using 2 µl of cDNA. GAPDH was quantified and used as an internal control to normalize tRNA expression levels. After the first amplification of cDNA, quantification of tRNA expression and copy number was performed by SNaPshot Sequencing, as described in (Santos et al., 2018).

### Cellular Viability Assay

1.5 × 10^5^ cells/well were seeded in a 24-well plate. After 48 h, cells were detached and equal volumes of cell suspension and trypan blue were mixed. Finally, cell viability (%) was obtained by counting the live and death cells using a TC10Tm Automated Cell Counter (Bio-Rad). This assay was performed with triplicates and repeated three times.

### Cellular Proliferation Assay

To evaluate cellular proliferation, we used a colorimetric immunoassay ELISA, based on the measurement of BrdU incorporation during DNA synthesis (Roche, Cat.11647229001), following the manufacturer’s instructions. 5 × 10^3^ cells/well were plated in a 96-well and analysis was performed after 48 h.

### Anchorage-Dependent Colony Formation Assay

300 cells per condition were seeded in 60 mm dishes and maintained in culture for 2 weeks (BEAS-2B derived cell lines) or 9 days (H460 Mock). The colonies were then fixed using ice cold methanol and incubated at −30°C for 30 min. Methanol was removed and a solution of 0.1% crystal violet in 20% methanol was added and the plates were incubated at room temperature for 30 min. Plates were washed with H_2_O milliQ to remove excess dye and the colonies were counted. This assay was performed in triplicates and repeated four times.

### Protein Synthesis Rate Determination

To determine protein synthesis rate, we used a non-reactive fluorescence-activated cell sorting-based assay called SUnSET, with few modifications. 1 × 10^6^ cells were plated in 60 mm Petri dishes and after 48 h, 10% v/v puromycin (Sigma Aldrich) was added to each plate. Cells were then incubated for 15 min. Total protein lysates were obtained from cells with Lysis Buffer (0.5% Triton X-100, 50 mM HEPES, 250 mM NaCl, 1 mM DTT, 1 mM NaF, 2 mM EDTA, 1 mM EGTA, 1mM PMSF, 1 mM Na3VO4 supplemented with a cocktail of protease inhibitors (Roche). Cells were sonicated with a probe sonicator in five pulses of 5 s. After centrifugation at 16000 *g* for 30 min, protein in the supernatants was quantified using the BCA assay (Thermo Fisher Scientific). 100 µg of protein was denaturated with loading buffer (6x) at 95°C for 5 min, resolved in 10% SDS-PAGE and blotted onto nitrocellulose membranes (0.2 µm) (GE Healthcare Life Sciences). Anti-puromycin antibody, clone 12D10 (kindly given by Doctor Philippe Pierre) was used (dilution 1:2,500) to detect the incorporation of puromycin into proteins. IRDye800 goat anti-mouse secondary antibody (Li-cor Biosciences, Cat.400-33) was used (1:10000 dilution) and detected in an Odyssey Infrared Imaging System (Licor Biosciences). Membranes were also probed with Anti-GAPDH (Santa Cruz Biotechnology, Cat. sc-25778) (dilution 1:1,000) as a loading control.

### Immunoblots

Total protein lysates were obtained with the same extraction method used with the SUnSET assay. 40 µg of protein were immunoblotted onto nitrocellulose membranes with antibodies against eIF2α (1:1,000; Cell signalling, Cat.9722); phospho-eIF2*α* (1:1,000; Abcam, Cat.ab4837); BIP (1:1,000; Stress Marq Biosciences, Cat.SPC-180); GADD34 (1:1,000; ThermoFisher Scientific, Cat# PA5-30486); phospho-ATF4 (1:1,000; tebu-bio, Cat. BS4020); ATF4 (1:1,000; tebu-bio, Cat. BS1026); anti-ubiquitin (1:1,000; Sigma-Aldrich, Cat. U0508) and *ß*-tubulin (1:1,000; Invitrogen, Cat.32–2,600). IRDye680 goat anti-rabbit or IRDye800 goat anti-mouse secondary antibodies (1:10,000; Li-cor Biosciences, Cat.926-68071 [rabbit], Cat.400-33 [mouse]) were used and the signal was detected using an Odyssey Infrared Imaging system (Li-cor Biosciences).

### Proteasome Activity Assay

Proteasome activity was assessed as in (Varanda et al., 2020). Briefly, 2 × 10^5^ cells were plated in 6-well plates. After 48 h cells were washed with 1%PBS and resuspended in 100 µl of Lysis Buffer, sonicated and centrifuged at 13,000 rpm for 10 min at 4°C. Twenty micrograms of protein were incubated with suc-LLVY-MCA (50 µM) (Sigma-Aldrich, Cat. S4939) in the presence or in the absence of the proteasome inhibitor MG132 (10 µM) (Sigma-Aldrich, Cat. SML1135). Substrate degradation was monitored every 5 min during 1 h at 37°C in a fluorescence-luminescence detector Synergy™ HT Multi-Mode Microplate Reader (Biotek). Proteasome activity was calculated by subtracting fluorescence emission at 0 min from fluorescence after 1 h relative to control (Mock).

### Tumor Induction Assay

Six-week-old male N:NIH(s)II:nu/nu nude mice were obtained previously from the Medical School, University of Cape Town in 1991 and then reproduced, maintained and housed at IPATIMUP Animal House, at the Medical Faculty of the University of Porto, in a pathogen-free environment under controlled conditions of light and humidity. Male nude mice, aged 6–8 weeks, were used for *in vivo* experiments. Animal experiments were carried out in accordance with the Guidelines for the Care and Use of Laboratory Animals, directive 2010/63/EU, and the experiments were submitted to internal ethical approval. To measure tumorigenic potential *in vivo*, BEAS-2B cell lines harboring the empty vector (Mock) and the tRNA^Ser^(AGA) gene were subcutaneously injected in the dorsal flanks using a 25-gauge needle with 4.5 × 10^6^ cells of each cell line. A total of four mice were used. Each mouse was injected in the left flank with cells carrying the Mock plasmid and in the right flank with cells carrying the tRNASer(AGA) plasmid. Mice were weighed, and tumor width and length were measured with calipers once per week. Mice were humanely euthanized after 3 months. Tumors and lungs, were collected, fixed in 10% buffered formalin, paraffin embedded and then sectioned for histological examination and stained for hematoxylin and eosin.

### Statistical Analysis

For all the assays, except for the *in vivo* experiments, our data represents three independent biological replicates and three to four independent experiments. Average values are usually shown and error bars represent the standard error of the mean (SEM). Statistical significance was determined using Student’s t-test or One-way ANOVA with Holm Sidak’s post-test. *p* values < 0.05 were considered statistically significant.

## Results

### Increased Abundance of tRNA^Ser^(AGA) has a Positive Impact on Cellular Fitness

**FIGURE 1:**
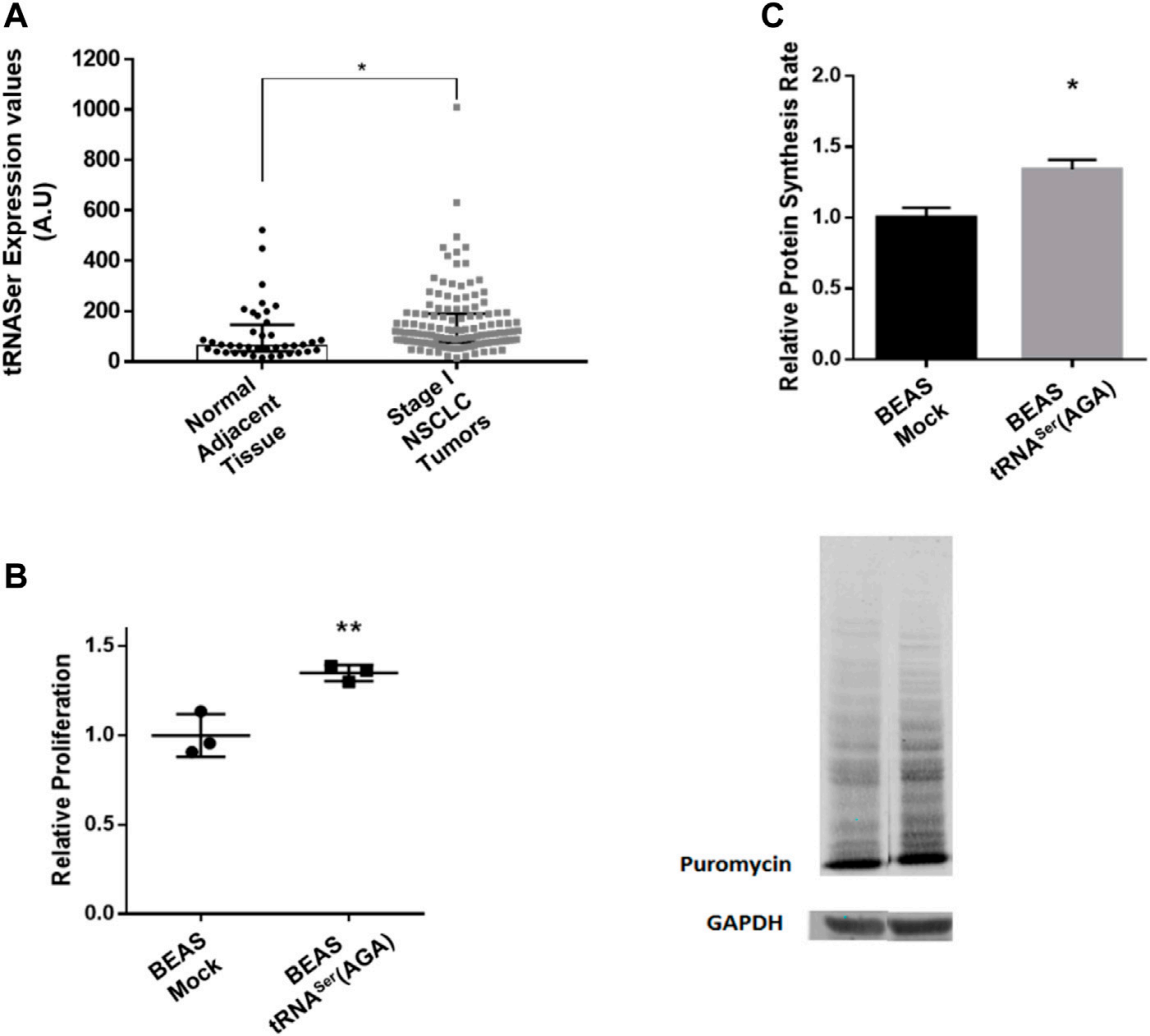
Overexpression of tRNA^Ser^ increases cell proliferation and protein synthesis rate. **(A)** tRNA-Ser-AGA-2-1 expression levels are increased in Stage I tumors when compared to normal adjacent tissue. Data was analyzed with Student’s t-test and significant *p* values is shown (*p < 0.05) **(B)** To evaluate cellular proliferation, 5 × 10^3^ cells/well were plated in 96-well plates and incubated for 48 h prior to measuring BrdU incorporation during DNA synthesis, using a colorimetric immunoassay. Relative proliferation of BEAS-2B derived cell lines–The overexpression of tRNA^Ser^ increased cellular proliferation capacity *in vitro.* **(C)** tRNA^Ser^(AGA) upregulation increased protein synthesis rate in normal lung cell lines. Protein synthesis rate was estimated by immunoblot against puromycin. GAPDH served as a protein loading control. Graphics depict average ± SEM of *n* = 3, with three technical replicates. Data was analyzed with One-way ANOVA and Holm Sidak’s post-test and significant *p* values are shown (*p < 0.05; **p < 0.01).

Our previous results showed that tRNA-Ser-AGA-2-1 is involved in tumor growth and UPR activation, but it was unclear whether deregulation of its expression is involved in tumor establishment. To clarify this issue, we used Non-Small Cell Lung Cancer cells as a model for our studies. Using the data from the TCGA repository and the tRiC database (Zhang et al., 2020), we assessed the expression levels of tRNA-Ser-AGA-2-1 (hereafter tRNA^Ser^(AGA)) in 130 Stage I lung tumors and 40 normal adjacent lung tissues. In normal adjacent tissue the median expression value for tRNA^Ser^(AGA) was 63.945 Arbitrary Units (A.U.), while in Stage I tumors, the median expression value was 114.595 A.U. (Figure 1A). This represented a 1.79-fold increase in the expression of tRNA^Ser^(AGA) in tumors relative to normal adjacent tissue (*p < 0.05*).

To evaluate the consequences of increasing the tRNA^Ser^(AGA) abundance in normal lung cells, we used a normal bronchus epithelial cell line (BEAS-2B). Three independent stable cell lines were established expressing either the tRNA^Ser^(AGA) or the Mock vector (Supplementary Figures 1A,B). To exclude the hypothesis that the phenotypes observed were transfection specific, we established three independent stable cell lines for each condition (Supplementary Figures 1A,B). The tRNA^Ser^(AGA) was upregulated on average 1.6-fold (*p* < 0.05), relative to the Mock vector (Supplementary Figure 1C), mimicking the upregulation observed in Stage I tumors relative to normal tissue (Supplementary Figure 1A). Similarly to other published results (Santos et al., 2018; Varanda et al., 2020), stable upregulation of the tRNA^Ser^(AGA) did not affect the viability of BEAS-2B cells (Supplementary Figure 1D).

Upregulation of tRNA^Ser^(AGA) increased cell proliferation by 1.35-fold, relative to the Mock (*p* < 0.01) (Figure 1B), raising the hypothesis that protein synthesis rate could be elevated to sustain proliferation. Using the SunSET technique to determine the incorporation of puromycin into proteins, we saw a 1.34-fold increase in protein synthesis rate in the cell line expressing the tRNA^Ser^(AGA) (*p* < 0.05) (Figure 1C). Therefore, the increased cell proliferation is accompanied by an increase in protein synthesis rate in these cells.

### Upregulation of tRNA^Ser^(AGA) Induces the Unfolded Protein Response and *UPS*

**FIGURE 2:**
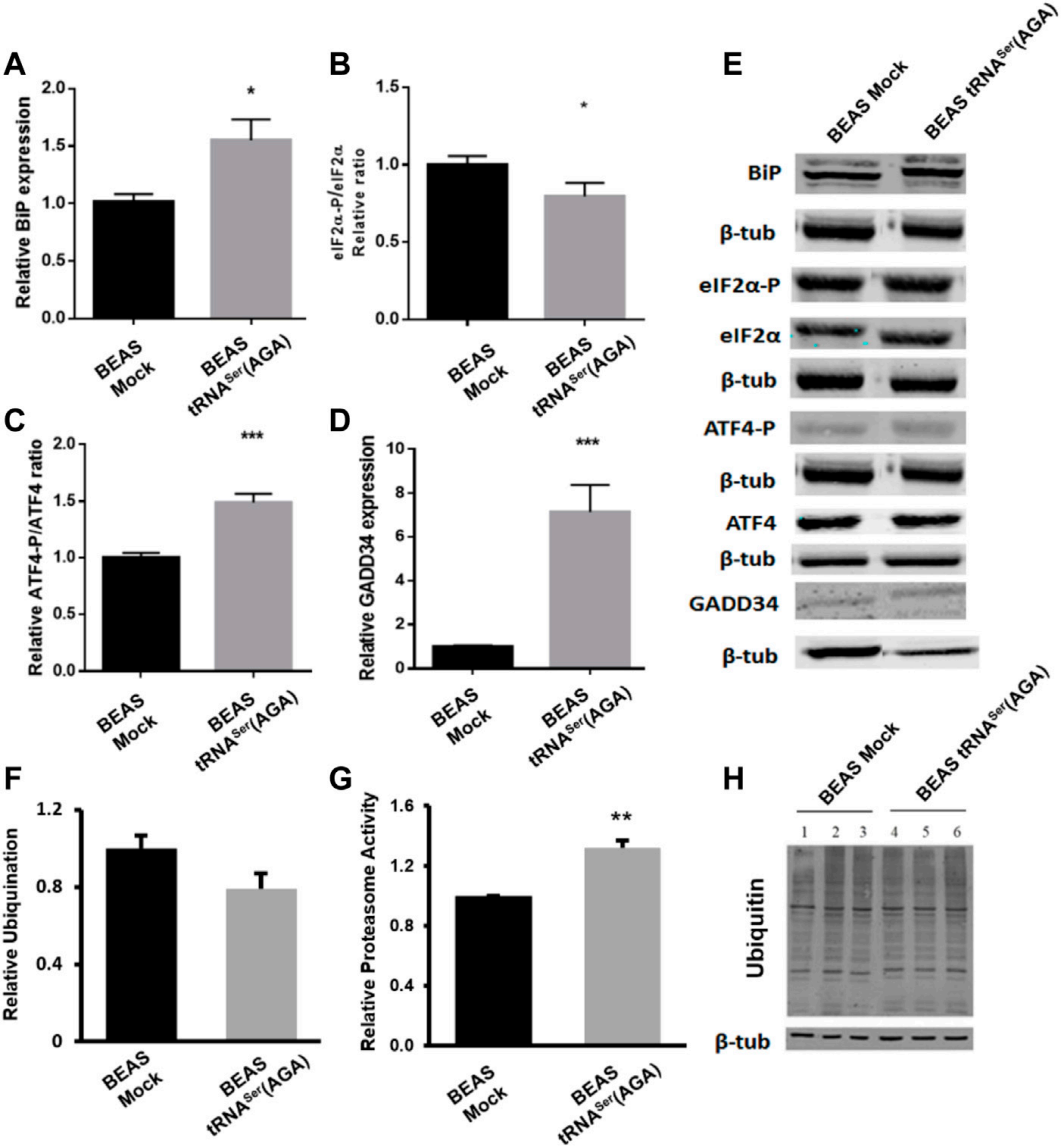
UPR and UPS deregulation induced by recombinant tRNASer(AGA). **(A)** Expression of tRNA^Ser^(AGA) increases BiP expression in BEAS-2B cells. **(B)** Levels of eIF2*α*-P were decreased in cells with tRNA^Ser^(AGA). **(C)**tRNASer(AGA) expression in BEAS-2B cell line increased the activation of ATF4 transcription factor. **(D)** Expression of GADD34 was increased in BEAS-2B cells expressing the recombinant tRNA^Ser^(AGA). **(E)** Immunoblots using antibodies against BiP, eIF2*α*-P, eIF2*α*, ATF4-P, ATF4 and GADD34. Β-tub served as protein loading control. Graphics depict average ±SEM (*n* = 3), with three technical replicates. **(F)** No differences are observed between BEAS-2B Mock and tRNA^Ser^(AGA) cells regarding ubiquitin levels. Graphic represents average ±SEM of five independent experiments. **(G)** Upregulation of tRNA^Ser^(AGA) in BEAS-2B increases proteasome activity by 1.32-fold. Graphic depicts average ±SEM (*n* = 4), with three technical replicates. **(H)** Expression of ubiquitin. *ß*-tubulin represents the internal control. Total protein was extracted from BEAS-2B Mock cells (lanes 1, 2 and 3) and BEAS-2B tRNA^Ser^(AGA) (lanes 4, 5 and 6). Image represents results from one independent experiment with all technical replicates. Data was analyzed with One-way ANOVA and Holm Sidak’s post-test and significant *p* values are shown (*p < 0.05; **p < 0.01; ***p < 0.001).

As the PERK branch of the UPR is important for tumor establishment and BiP deregulation was already observed in pre-malignant lung lesions, we studied their deregulation in our model. BiP was upregulated 2.8-fold in BEAS-2B cells expressing the tRNA^Ser^(AGA) relative to Mock cells (*p* < 0.05) (Figures 2A,E), while eIF2*α* phosphorylation levels decreased 0.21-fold (*p* < 0.05) (Figures 2B,E). These results are positively correlated with the increased protein synthesis rate observed in Figure 1C, and supported by our previous published data (Santos et al., 2018). This result might seem contrary to PERK activation since its activation leads to eIF2*α* phosphorylation. However, this event starts a negative feedback loop to dephosphorylate eIF2*α* and restore global protein synthesis through ATF4 activation and GADD34 expression (Harding et al., 2000b; Novoa et al., 2001). Therefore, we investigated if this was the mechanism responsible for eIF2*α* dephosphorylation in tRNA^Ser^(AGA) cells and indeed, we detected an increase of 1.49-fold in ATF4 activation relative to the mock control (*p* < 0.01) (Figures 2C,E). Additionally, the GADD34 regulatory subunit, which directs the PP1α phosphatase to eIF2α, was upregulated 6.86-fold (*p* < 0.05). (Figures 2D,E). These results show that tRNA^Ser^(AGA) upregulation activates the UPR, namely the PERK branch of the UPR, which is consistent with increased protein synthesis and cellular proliferation.

Furthermore, BiP is involved in Endoplasmic Reticulum Associated Degradation (ERAD) targeting non-glycosylated proteins for proteasome degradation (Ushioda et al., 2013). Despite no alteration was found in ubiquitylation levels (Figures 2F,H), upregulation of tRNA^Ser^(AGA) lead to an increase of 1.32-fold in proteasome activity (Figure 2G).

Interestingly, BiP upregulation, lower levels of eIF2*α* and proteasome activation are important characteristics of cellular transformation and lung premalignant lesions.

### tRNA^Ser^(AGA) Upregulation Increases the *in vitro* Transformation Potential of BEAS-2b

**FIGURE 3:**
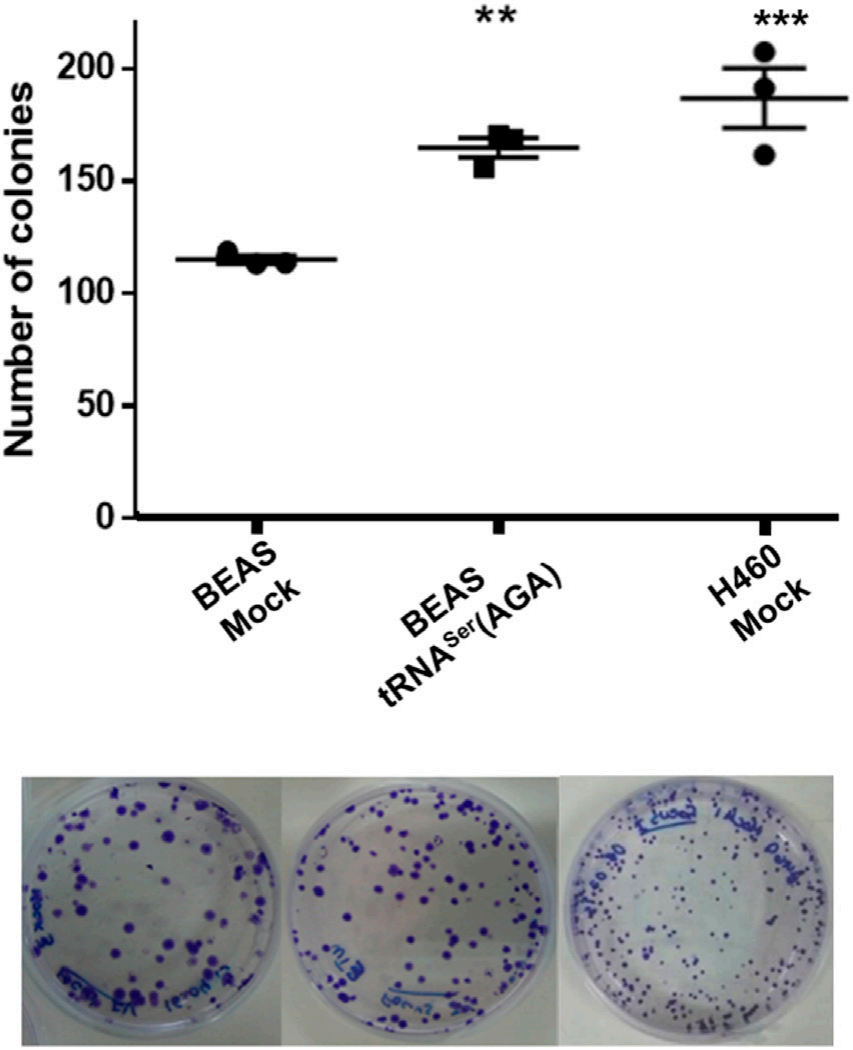
tRNA^Ser^(AGA) upregulation increases the transforming potential of normal cells *in vitro*. Upper panel, quantification of colonies in three biological replicates per condition. Lower panel, Representative anchorage-dependent colony formation plates used for quantification.

Upregulation of tRNA^Ser^(AGA) was also able to increase *in vitro* the number of colonies of BEAS-2B cells to levels identical to those of the tumoral cell line H460, in an anchorage-dependent colony formation assay (1.43-fold *vs*. BEAS-2B Mock cell line (*p* < 0.01)) (Figure 3). Interestingly, the number of BEAS-2B tRNA^Ser^(AGA) colonies obtained with this assay in different transfections was very similar, indicating that this phenotype is not transfection-specific (Figure 3). These results suggest that deregulation of tRNA^Ser^(AGA) expression alone is sufficient to increase the transforming potential of normal immortalized cells.

### tRNA Deregulation Impacts Tumor Establishment *in vivo*

**FIGURE 4:**
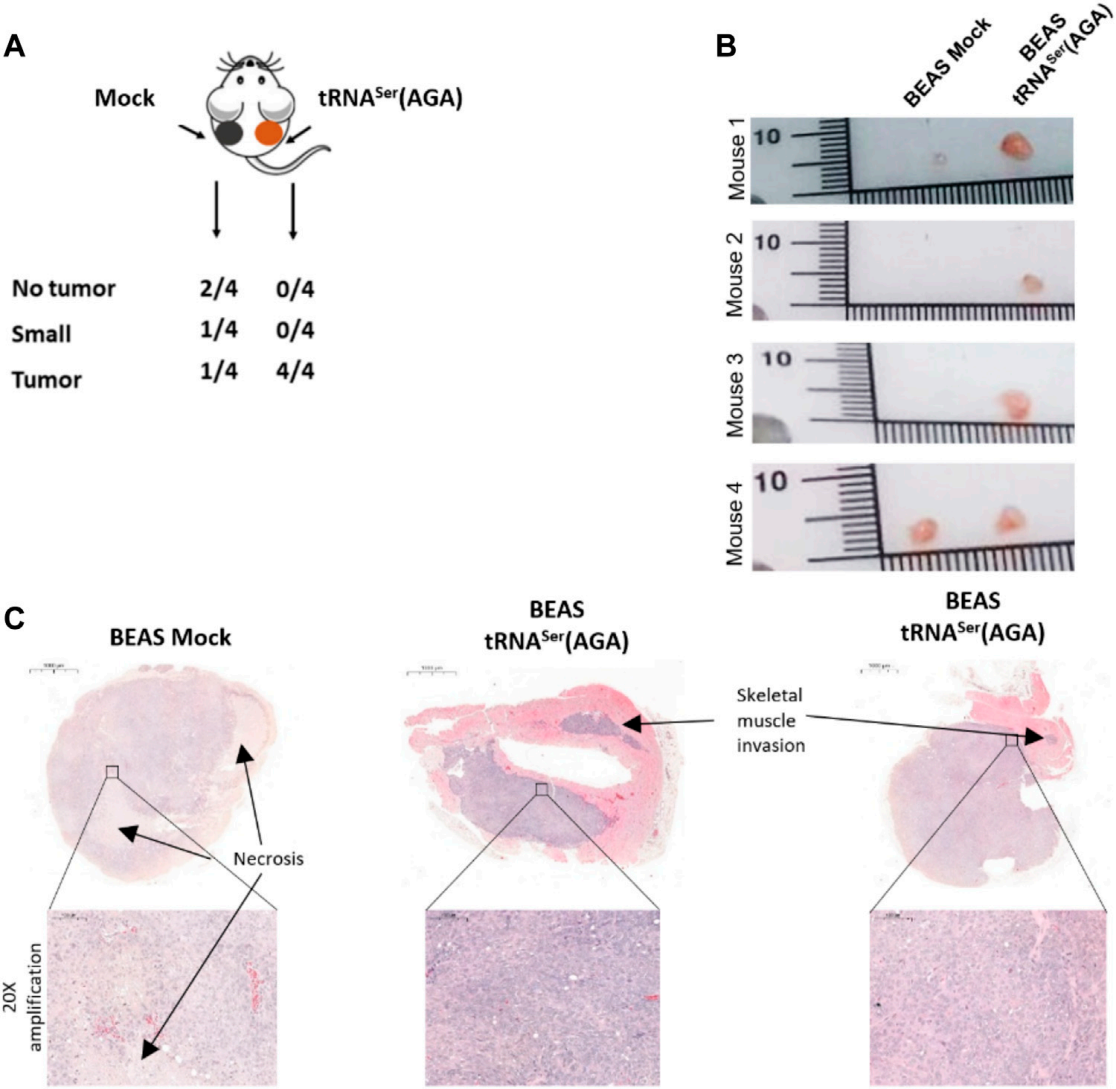
Effect of tRNA deregulation in tumor initiation. **(A)** All animals inoculated with BEAS-2B cells harboring the recombinant tRNA^Ser^(AGA) developed a tumor, whereas only 50% of the mice had BEAS-2B Mock tumors of small size. **(B)** Macroscopy imagens of Mock and tRNA^Ser^(AGA)-derived tumors inoculated in the same animal **(C)** H&E of Mock and tRNA^Ser^(AGA)-derived tumors. Upper part, Digitalization of whole tumors with indication (∼3.3x amplification) of Skeletal Muscle invasion and Necrotic areas. Bottom part. 20x amplification of indicated tumoral areas.

To assess whether tRNA^Ser^(AGA) upregulation impacted tumor establishment, we inoculated 4.5 × 10^6^ BEAS-2B-derived cells [tRNA^Ser^(AGA)] in the right dorsal flank of four mice and the control cells (Mock) in the corresponding left dorsal flank of every mice. The experiment was terminated 3 months post-inoculation. All mice (4/4) developed tRNA^Ser^(AGA)-derived tumors in the right dorsal flank, whereas only two animals (2/4) developed tumors in the left flank inoculated with Mock cells. The two Mock tumors were extremely small, whereas the four tRNA^Ser^(AGA)-derived tumors were several fold greater in size (Figures 4A,B). Both Mock- and tRNA^Ser^(AGA)-derived tumors’ histology resembled grade III NSCLC tumors, exhibiting accentuated cytologic atypia, but no pleiomorfism, mucin presence, keratin or glandular structures, that would allow further stratification (Figure 4C). Pathology analysis of tRNA^Ser^(AGA)-derived tumors revealed signs of malignancy, such as invasion of skeletal muscle tissue, as well as increased mitotic index (Figure 4C). Therefore, despite their similar histology, Mock and tRNA^Ser^(AGA)-derived tumors present different pathological findings which render the later more aggressive. This supports the hypothesis that tRNA^Ser^(AGA) upregulation is relevant for tumor establishment and progression *in vivo*. In conclusion, as tumors also developed from BEAS Mock cells, we cannot infer this tRNA to be a primary trigger for tumor initiation, but rather a factor that promotes and/or accelerates malignant phenotypes.

## Discussion

Our work shows that tRNA-Ser-AGA-two to one is overexpressed in Stage I NSCLC by 1.79-fold when compared to normal adjacent tissue. Upregulation of tRNA^Ser^(AGA) in normal human bronchial cells to a similar extent, boosted protein synthesis and increased cellular proliferation. This event also increased transforming ability *in vitro* and tumor development *in vivo,* with tRNA^Ser^(AGA)-derived tumors presenting with obvious pathological signs of aggressiveness. These results are in line with previous reports of Pavon and others, showing that mild upregulation of tRNAiMet is sufficient to increase metabolic activity and proliferation rates (Pavon-Eternod et al., 2013). Wang et al., further suggested that tRNAiMet can be an initiation factor in breast cancer (Wang et al., 2018). These data is herein supported as upregulation of tRNA^Ser^(AGA) increased colony formation capacity to levels that are similar to those of H460 tumoral cells (De Marco et al., 2015), and increased tumor establishment with aggressive features *in vivo*.

Our data also showed that of tRNA^Ser^(AGA) upregulation activated the PERK branch of the UPR, and decreased the levels of phosphorylated eIF2*α*, which has been associated to cellular transformation (Perkins and Barber, 2004). This puzzling result may be explained by the strong upregulation (6.86-fold) of the PP1 alpha phosphatase subunit GADD34, which potentially underlines the increased protein synthesis rate observed in this cell line. Since tRNA^Ser^(AGA) upregulation increased levels of transcription factor ATF4 activation, it is likely that genes relevant for adaptation under stressful conditions like the ones imposed by tumor microenvironment (hypoxia, oxidative stress and nutrient deprivation) may be upregulated, besides GADD34 transcription, (Fels and Koumenis, 2006; Lewerenz and Maher, 2009; Ye et al., 2010). Moreover, BiP protein, which has been detected in head and neck cancer initiating cells and in lung pre-malignant lesions (Wu et al., 2010; Kim et al., 2012), was upregulated in the tRNA^Ser^(AGA) cells. Using a higher number of mice in future studies, would allow for the collection of enough tumor material to establish the relevance of this mechanism in tumor establishment by upregulation of tRNA^Ser^(AGA) in BEAS cells.

The increased proteasome activity in cells with tRNA^Ser^(AGA) upregulation also correlates with the results obtained for cellular proliferation and increased tumorigenic potential, since this organelle plays a part in both processes (Guo et al., 2016; Walerych et al., 2016). Transformed cells rely to proteasome activity to thrive (Tsvetkov et al., 2018) and combination therapy strategies using proteasome inhibitors have been successful in treating several tumors (Mimnaugh et al., 2004). Therefore, it would be interesting to explore the effects of proteasome inhibition in our model in future studies.

Despite the low number of mice used in our *in vivo* pilot assay, our data indicate that upregulation of tRNA^Ser^(AGA) foster tumor establishment and growth of tumors mimicking grade III NSCLC. This was expected, since tumors arising in the bronchus often develop as NSCLC (Ferone et al., 2020). Importantly, tRNA^Ser^(AGA)-derived tumors showed signs of increased aggressiveness, such as increased mitotic index and skeletal muscle invasion, which were absent from Mock tumors.

Taken together, our results show that even a modest upregulation of the human tRNA^Ser^(AGA) has a positive impact on the fitness of normal lung epithelial cells, promotes changes in the UPR similar to those observed in pre-malignant lesions, and is sufficient to increase transforming potential *in vitro* and tumor establishment *in vivo*. tRNA levels are known to be tissue specific to match tissue gene expression profiles (Dittmar et al., 2006). As cellular stress trigger gene expression changes, global tRNA expression has to be adjusted to the stress specific mRNA pools to ensure adequate matches between codons and anticodons for efficient and accurate mRNA translation (Dittmar et al., 2006; Gingold et al., 2012). Interestingly, recent works show that changes in tRNA pools can reprogramme gene expression, in ways that promote invasion and metastization (Goodarzi et al., 2016). It would be interesting to clarify in future studies, if tRNA^Ser^(AGA) upregulation has similar effects on BEAS-2B cells growing for longer periods of time *in vivo*.

Our work highlights the importance of studying tRNA deregulation on early stage tumorigenesis, as it improves tumor establishment and fosters aggressiveness features. tRNA levels may be a biomarker of malignancy potential and may be valuable tools for addressing tumor aggressiveness.

## Data Availability

The datasets analyzed for this study can be found in the (tRiC DATABASE) (https://hanlab.uth.edu/tRic/download/).
