# Supplementary material for: Upregulation of tRNA-Ser-AGA-2-1 Promotes Malignant Behavior in Normal Bronchial Cells
Source: Front Mol Biosci. 2022 May 2;9:809985. doi: 10.3389/fmolb.2022.809985 (PMC9108184; doi:10.3389/fmolb.2022.809985)
Supplement: Supplementary file 1 [file Presentation1.pdf]

All Western Blots used for protein quantification on Manuscript 809985 Upregulation of tRNA-Ser-AGA-2-1 promotes malignant behavior in normal bronchial cells

Puromycin Blots - Fig. 1C, bottom panel

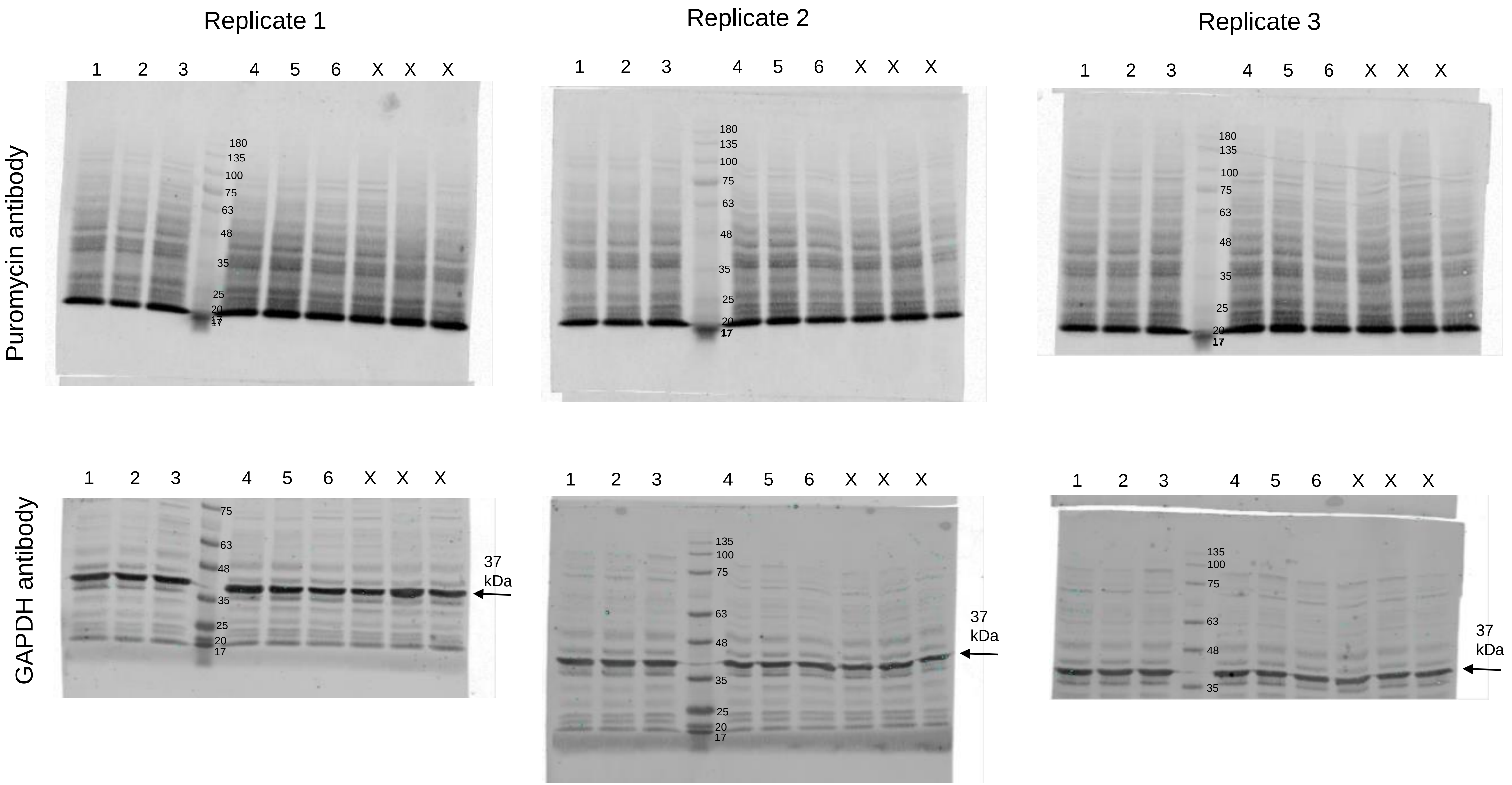

Legend: 1 - BEAS Mock 1; 2 - BEAS Mock 2; 3 - BEAS Mock 3; 4 - BEAS tRNASer 1; 5 - BEAS tRNASer 2; 6 - BEAS tRNASer 3; X – cell line containing a mutant tRNA not used in this manuscript

BIP Fig. 2E

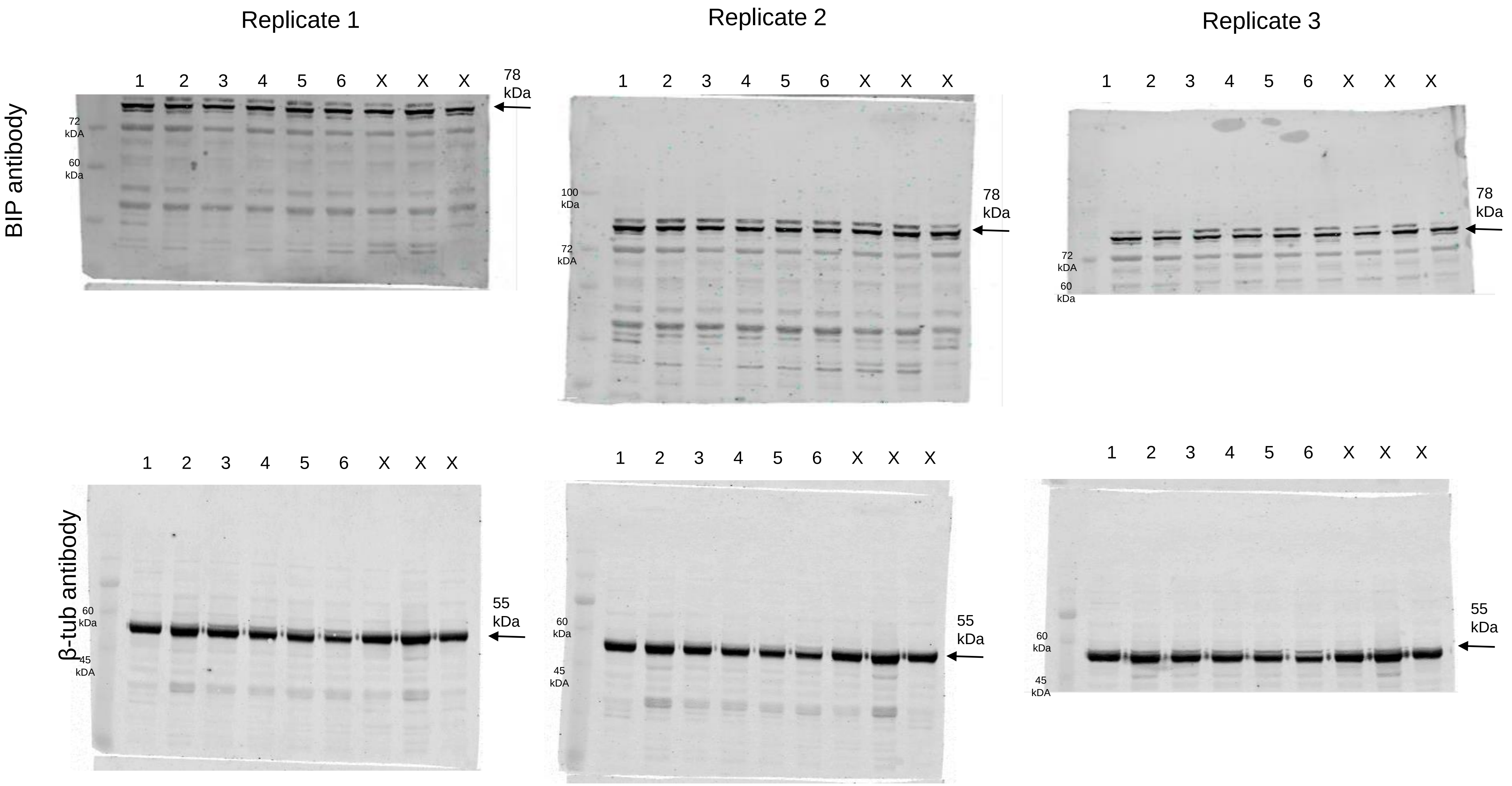

Legend: 1 - BEAS Mock 1; 2 - BEAS Mock 2; 3 - BEAS Mock 3; 4 - BEAS tRNASer 1; 5 - BEAS tRNASer 2; 6 - BEAS tRNASer 3; X – cell line containing a mutant tRNA not used in this manuscript

ATF4-P Fig. 2E

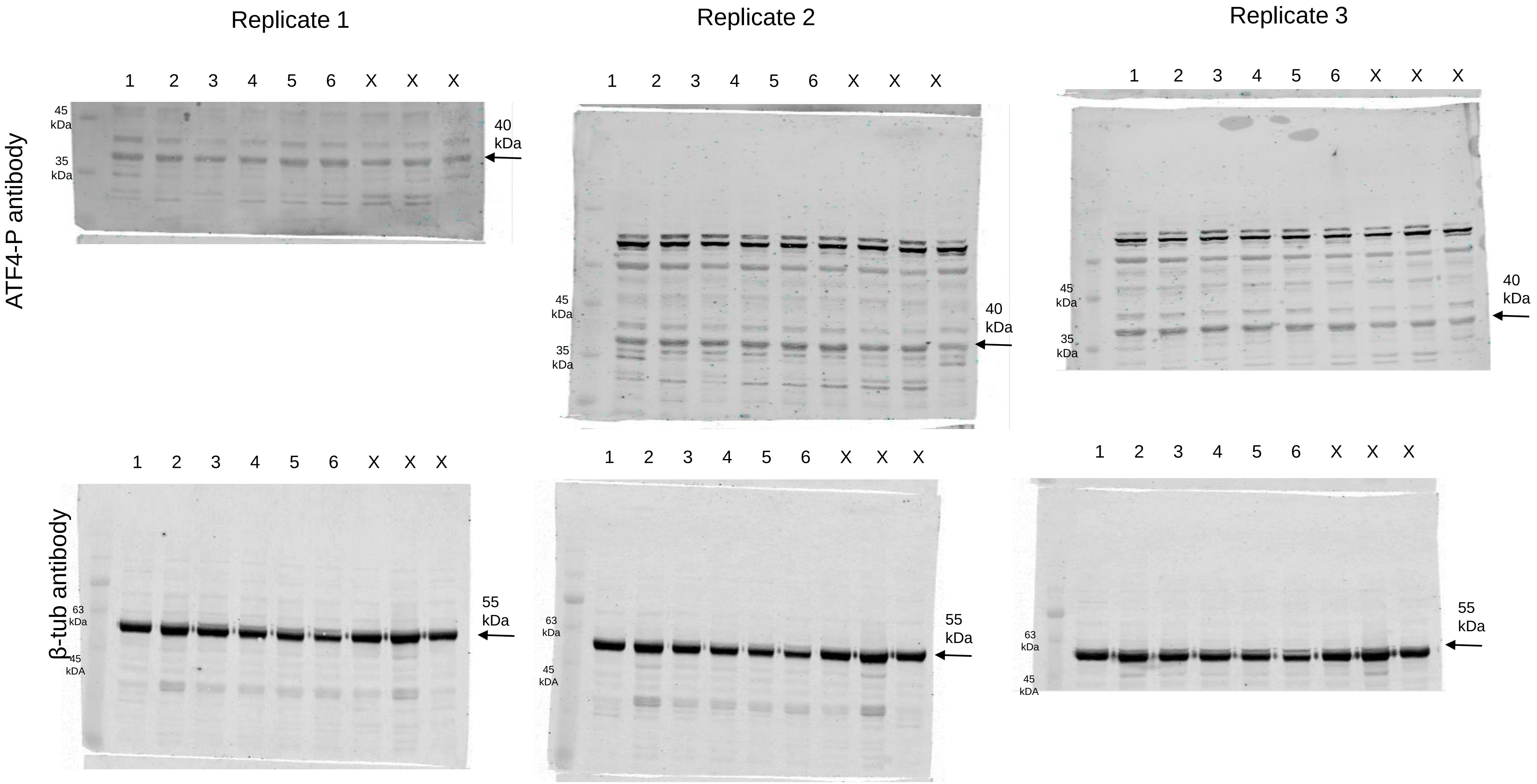

Legend: 1 - BEAS Mock 1; 2 - BEAS Mock 2; 3 - BEAS Mock 3; 4 - BEAS tRNASer 1; 5 - BEAS tRNASer 2; 6 - BEAS tRNASer 3; X – cell line containing a mutant tRNA not used in this manuscript

ATF4 total Fig. 2E

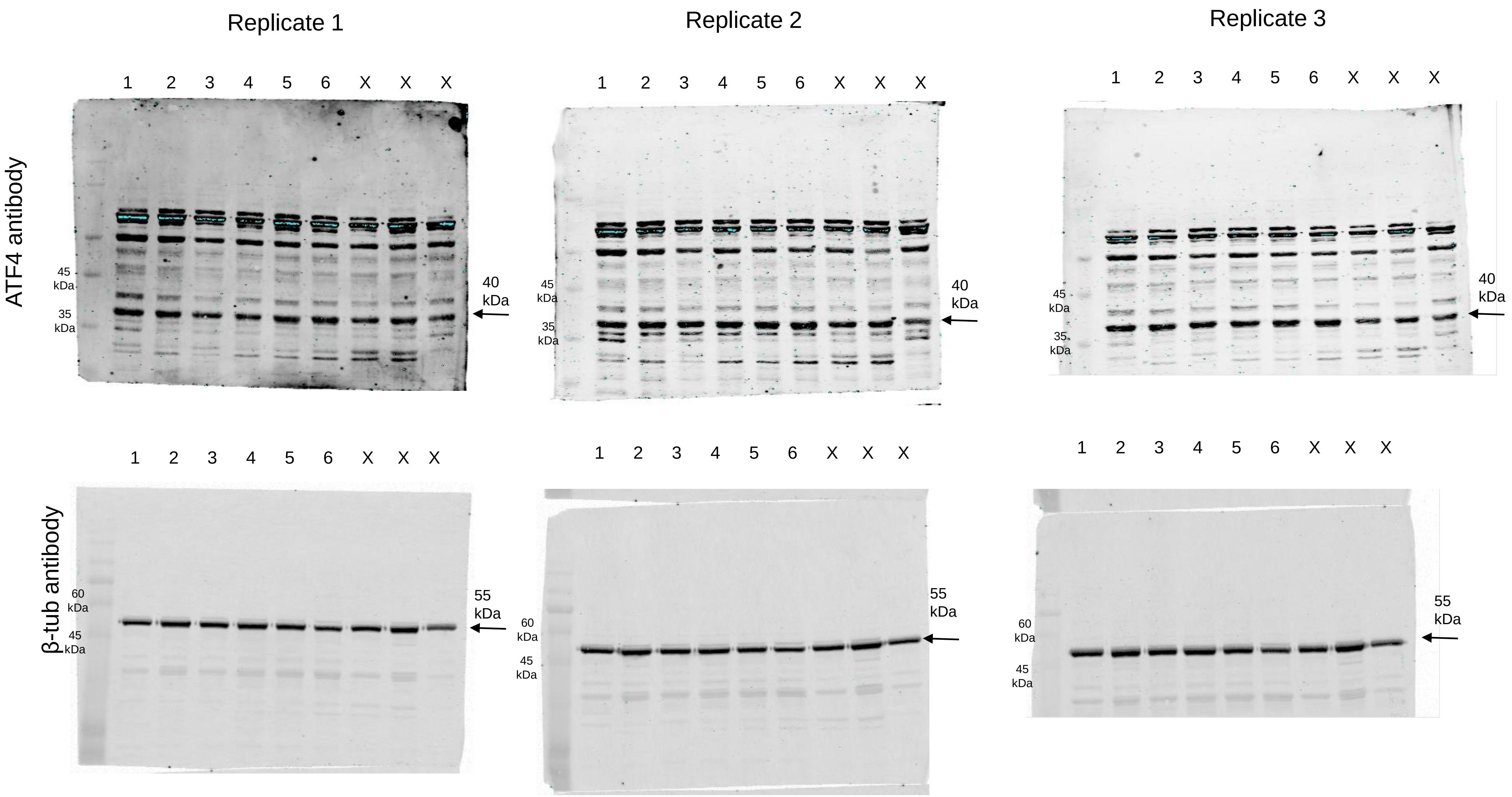

Legend: 1 - BEAS Mock 1; 2 - BEAS Mock 2; 3 - BEAS Mock 3; 4 - BEAS tRNASer 1; 5 - BEAS tRNASer 2; 6 - BEAS tRNASer 3; X – cell line containing a mutant tRNA not used in this manuscript

eiF2a Fig. 2E

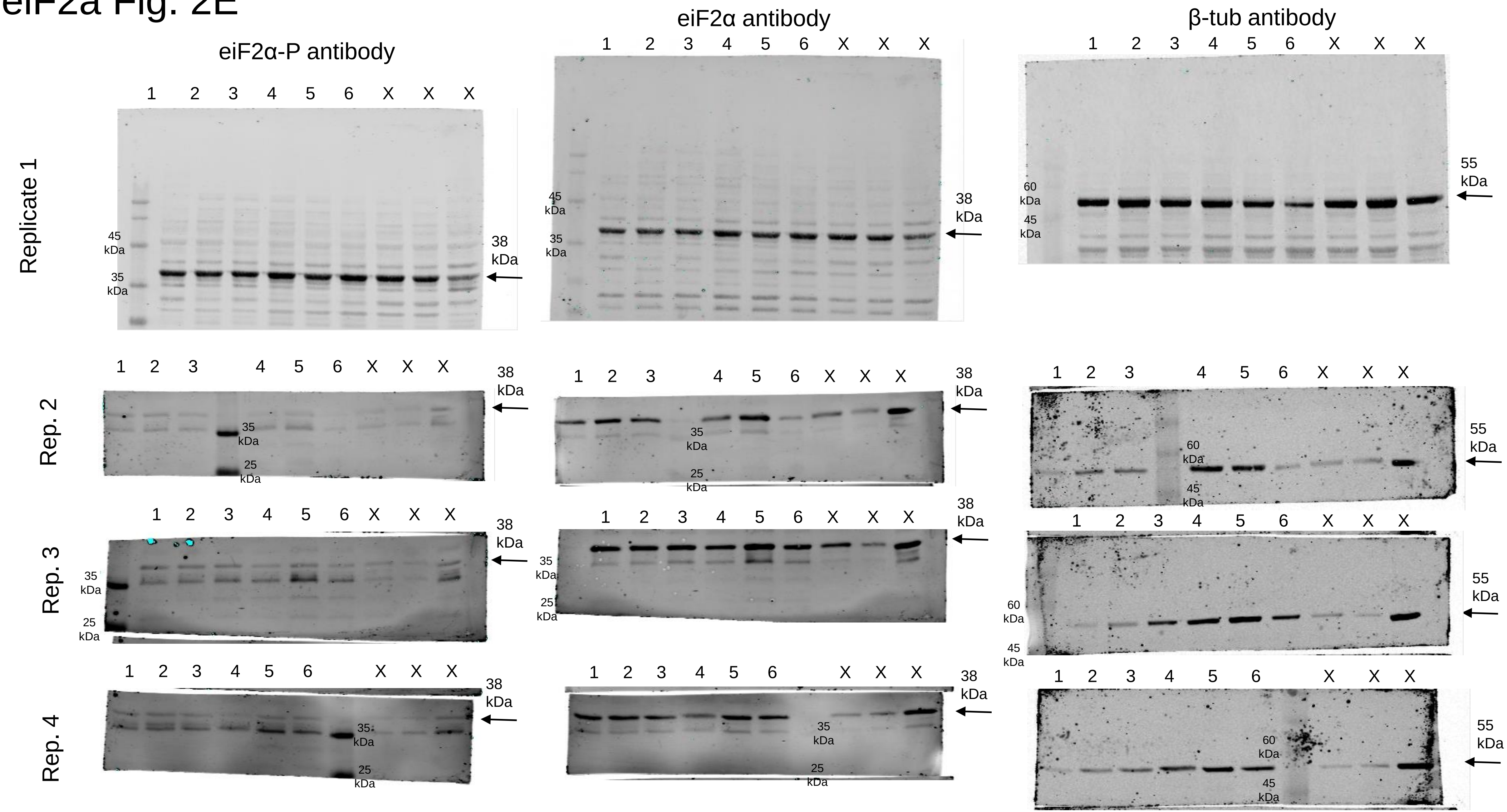

Legend: 1 - BEAS Mock 1; 2 - BEAS Mock 2; 3 - BEAS Mock 3; 4 - BEAS tRNASer 1; 5 - BEAS tRNASer 2; 6 - BEAS tRNASer 3; X – cell line containing a mutant tRNA not used in this manuscript

GADD34 Fig. 2E

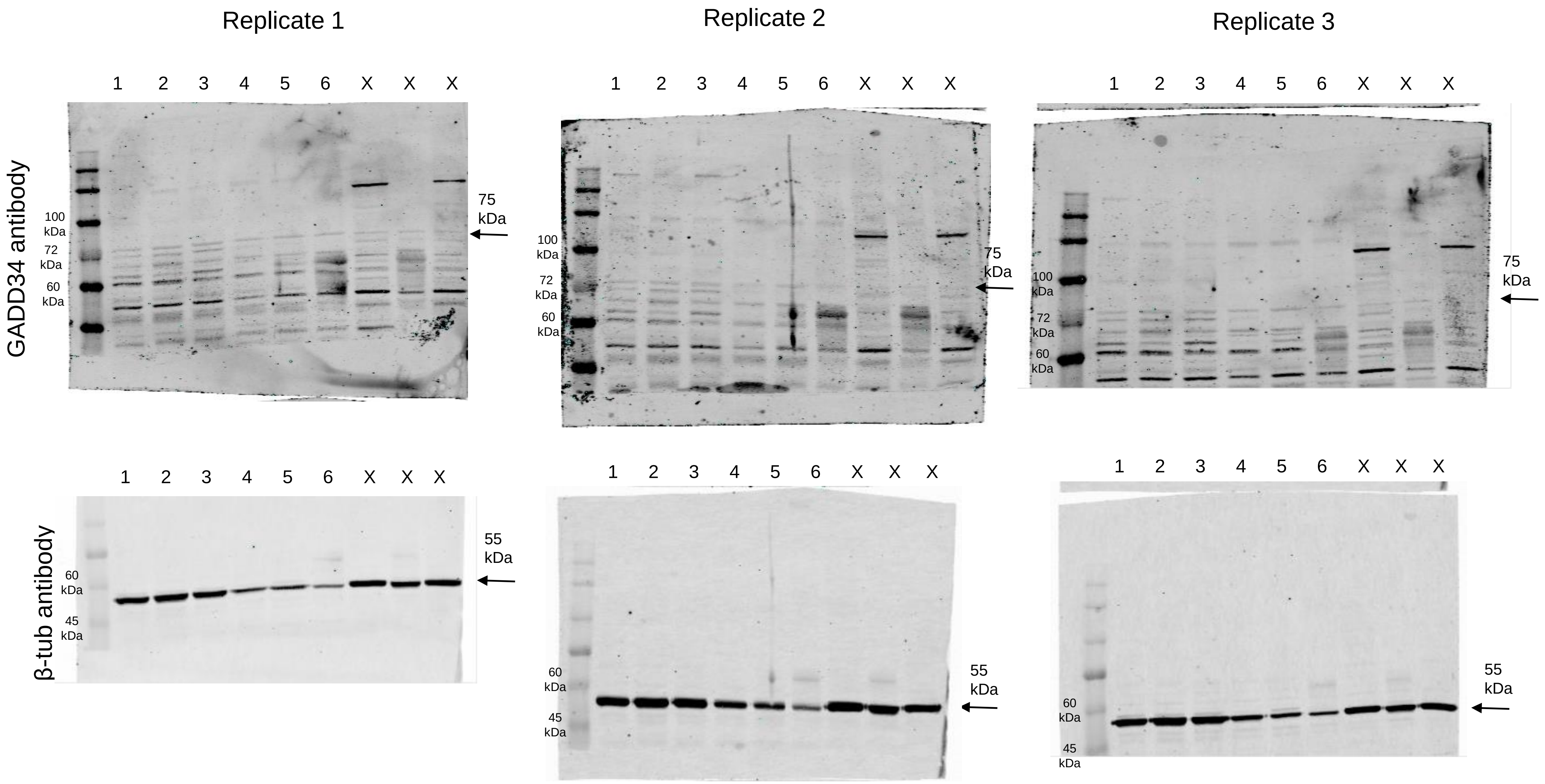

Legend: 1 - BEAS Mock 1; 2 - BEAS Mock 2; 3 - BEAS Mock 3; 4 - BEAS tRNASer 1; 5 - BEAS tRNASer 2; 6 - BEAS tRNASer 3; X – cell line containing a mutant tRNA not used in this manuscript
