# Supplementary figures and images for: Upregulation of tRNA-Ser-AGA-2-1 Promotes Malignant Behavior in Normal Bronchial Cells
Source: Front Mol Biosci. 2022 May 2;9:809985. doi: 10.3389/fmolb.2022.809985 (PMC9108184; doi:10.3389/fmolb.2022.809985)

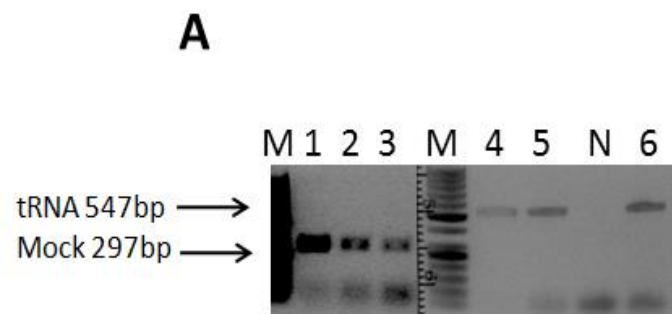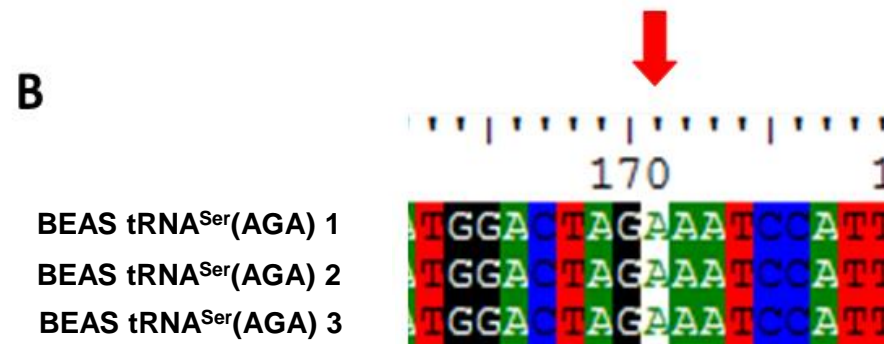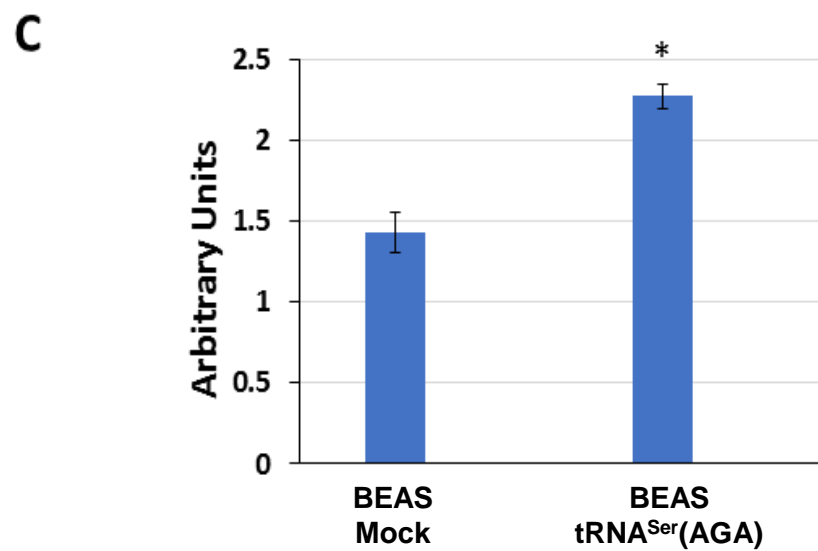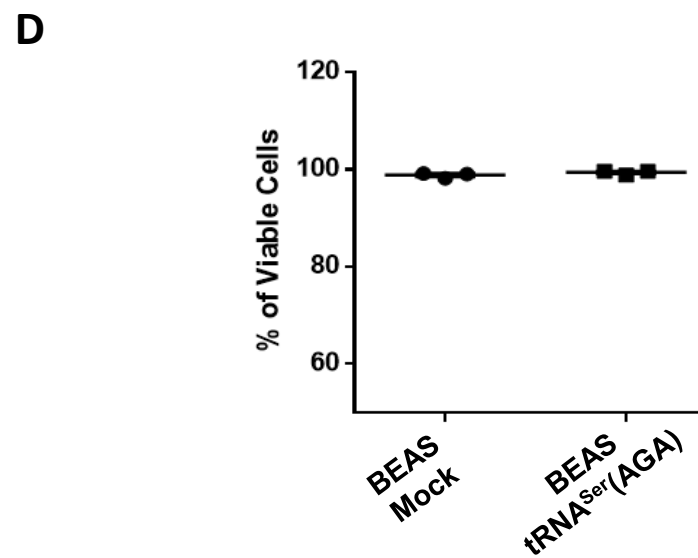

Supplement: Supplementary file 2 [file DataSheet1.PDF]
